# Acromioclavicular joint instability on cross-body adduction view: the biomechanical effect of acromioclavicular and coracoclavicular ligaments sectioning

**DOI:** 10.1186/s12891-022-05245-5

**Published:** 2022-03-23

**Authors:** Shimpei Kurata, Kazuya Inoue, Takamasa Shimizu, Mitsuyuki Nagashima, Hirakazu Murayama, Kenji Kawamura, Shohei Omokawa, Pasuk Mahakkanukrauh, Yasuhito Tanaka

**Affiliations:** 1 Department of Orthopedic Surgery, Nara Medical University, 840 Shijoutyou, Kashihara City, Nara, 634-5821 Japan; 2 Department of Hand Surgery, Nara Medical University, Kashihara, Nara, Japan; 3 Department of Anatomy Faculty of Medicine, Chiang Mai University, Chiang Mai, Thailand; 4 Excellence Center in Osteology Research and Training Center (ORCT), Chiang Mai University, Chiang Mai, Thailand

**Keywords:** Biomechanical study, Fresh frozen cadaver study, Acromioclavicular joint dislocation, Cross-body adduction view, Rockwood classification, Biomechanics

## Abstract

**Background:**

The acromioclavicular (AC) and coracoclavicular (CC) ligaments are important stabilizers of the AC joint. We hypothesized that AC and trapezoid ligament injuries induce AC joint instability and that the clavicle can override the acromion on cross-body adduction view even in the absence of conoid ligament injury. Accordingly, we investigated how sectioning the AC and CC ligaments contribute to AC joint instability in the cross-body adduction position.

**Methods:**

Six fresh-frozen cadaveric shoulders were used in this study, comprising five male and one female specimen, with a mean age of 68.7 (range, 51–87) years. The left side of the trunk and upper limb, and the cervical and thoracic vertebrae and sternum were firmly fixed with an external fixator. The displacement of the distal end of the clavicle relative to the acromion was measured using an electromagnetic tracking device. We simulated AC joint dislocation by the sequential resection of the AC ligament, AC joint capsule, and CC ligaments in the following order of stages. Stage 0: Intact AC and CC ligaments and acromioclavicular joint capsule; stage 1: Completely sectioned AC ligament, capsule and joint disc; stage 2: Sectioned trapezoid ligament; and stage 3: Sectioned conoid ligament. The superior clavicle displacement related to the acromion was measured in the horizontal adduction position, and clavicle overriding on the acromion was assessed radiologically at each stage. Data were analyzed using a one-way analysis of variance and post-hoc tests.

**Results:**

Superior displacement was 0.3 mm at stage 1, 6.5 mm at stage 2, and 10.7 mm at stage 3. On the cross-body adduction view, there was no distal clavicle overriding at stages 0 and 1, and distal clavicle overriding was observed in five cases (5/6: 83%) at stage 2 and in six cases (6/6: 100%) at stage 3.

**Conclusion:**

We found that AC and trapezoid ligament sectioning induced AC joint instability and that the clavicle could override the acromion on cross-body adduction view regardless of conoid ligament sectioning. The traumatic sections of the AC and trapezoid ligament may lead to high grade AC joint instability, and the distal clavicle may subsequently override the acromion.

## Introduction

Acromioclavicular (AC) joint dislocation is a common shoulder injury that accounts for 9%–10% of all shoulder injuries [1, 2]. The radiology-based Rockwood classification is commonly used to assess AC joint dislocation [3]. In this classification, types I and II are usually treated conservatively, whereas types IV to VI are usually treated surgically [4–7]. The management of type III injuries remains controversial [8]. Alexander [9] described the shoulder-forward view (the Alexander view), which can be used to assess AC joint injuries by thrusting the shoulder forward and evaluating acromion displacement anteriorly and inferiorly under the distal end of the clavicle. A similar cross-body adduction view was reported and used by Barnes et al. [10] to help identify whether the AC joint is stable. The International Society of Arthroscopy, Knee Surgery and Orthopaedic Sports Medicine (ISAKOS) [8] subclassified type III injuries as type IIIA and type IIIB based on cross-body adduction radiographs and recommended that patients with type IIIA injury who have a stable AC joint without clavicle overriding on the cross-body adduction view should be treated non-surgically and that patients with type IIIB injury with an unstable overriding joint should also be considered for surgical treatment because both coracoclavicular (CC) and AC ligaments are disrupted in this type of injury, suggesting that disruptions of the AC and CC ligaments influence AC joint dysfunction. It is important to evaluate how these disruptions affect the AC joint motion on the horizontal adduction position.

Anatomic studies revealed the unique orientation of the AC ligament [11] and unique attachment of the trapezoid and conoid ligaments at the coracoid process and clavicle [12, 13], which suggest the independent function of AC, trapezoid, and conoid ligaments. Regarding AC joint stability, biomechanical studies have revealed that the AC ligament stabilize the AC joint against vertical and horizontal translation [14–17]. The trapezoid ligament stabilizes the AC joint against horizontal translation [16, 18], and the conoid ligament stabilizes the AC joint against superior translation [14]. From these biomechanical facts [14–18], we guessed that the sequential section of AC and trapezoid ligaments induce the superior and posterior instability of the AC joint.

We hypothesized that the AC and trapezoid ligament injuries induce AC joint instability and that the clavicle can override the acromion on the cross-body adduction view without conoid ligament injury. Accordingly, this study aimed to investigate how sectioning the AC and CC ligaments can biomechanically contribute to AC joint instability in the cross-body adduction position.

## Materials and methods

### Specimen preparation

Fresh-frozen human cadavers of this study were provided by Department of Anatomy in Chiang Mai University. Six shoulders from six fresh-frozen cadavers, comprising five male specimens and one female specimen, were used in this study, with a mean age of 68.7 (range, 51–87) years. The specimens were prepared by thawing overnight at room temperature one day before the experiment. The specimens were subsequently sectioned above the first cervical vertebrae, below the sternum, and on the right side of the sternum and below the 11^th^ thoracic vertebrae. The left upper extremity was sectioned at the midshaft of the humerus, and a hole was drilled with a Kirschner wire with a diameter of 3 mm in the humerus near the attachment of the deltoid to pass a silk thread with a diameter of 2 mm that could be used to apply stress to the upper limb; tensile strength was approximately 400 N. All specimens were kept moist by spraying with normal saline during the experiment. Standard anteroposterior (AP) radiographs were obtained for each specimen, and no specimens had osteoarthritis at the sternoclavicular, AC, and glenohumeral joints.

**Fig. 1:**
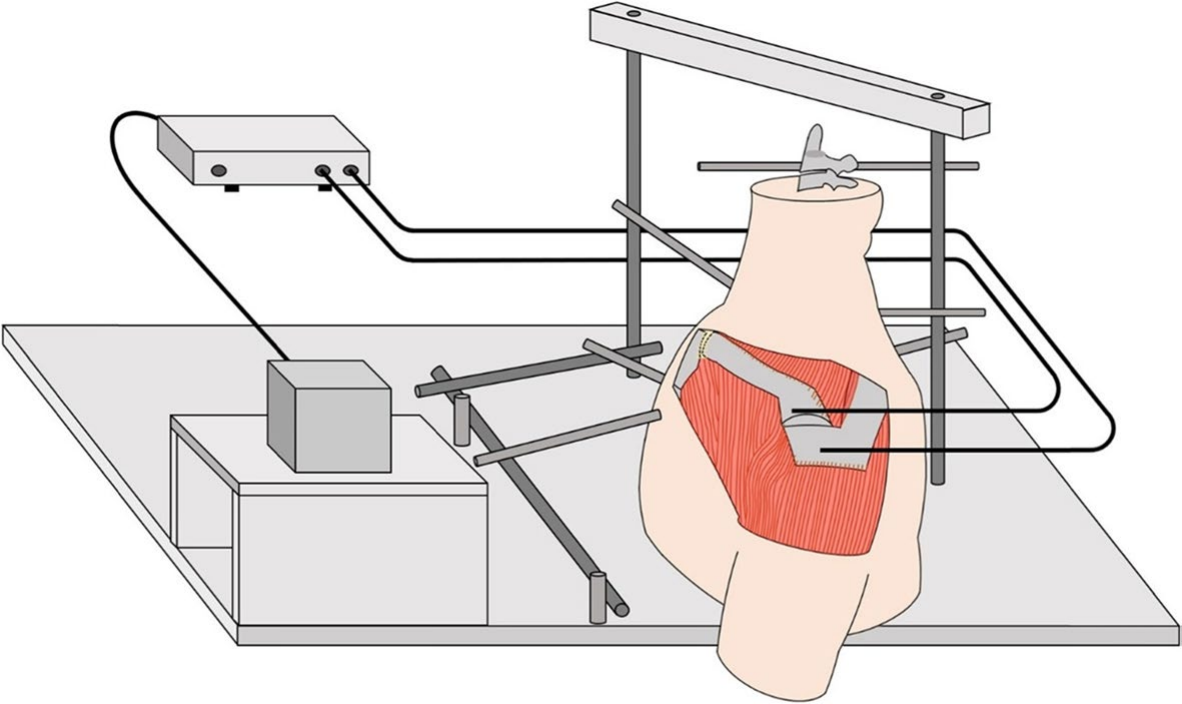
Experimental setup. The specimens were firmly fixed on a customized wooden jig with external fixators (Orthofix®; Japan Medicalnext Co., Ltd., Osaka, Japan). An electromagnetic tracking device (trakSTAR™; Ascension Technology Corporation, Shelburne, VT, USA) was used to measure the displacement of the distal end of the clavicle relative to the acromion. Sensors were inserted into the distal end of the clavicle and acromion

**Fig. 2:**
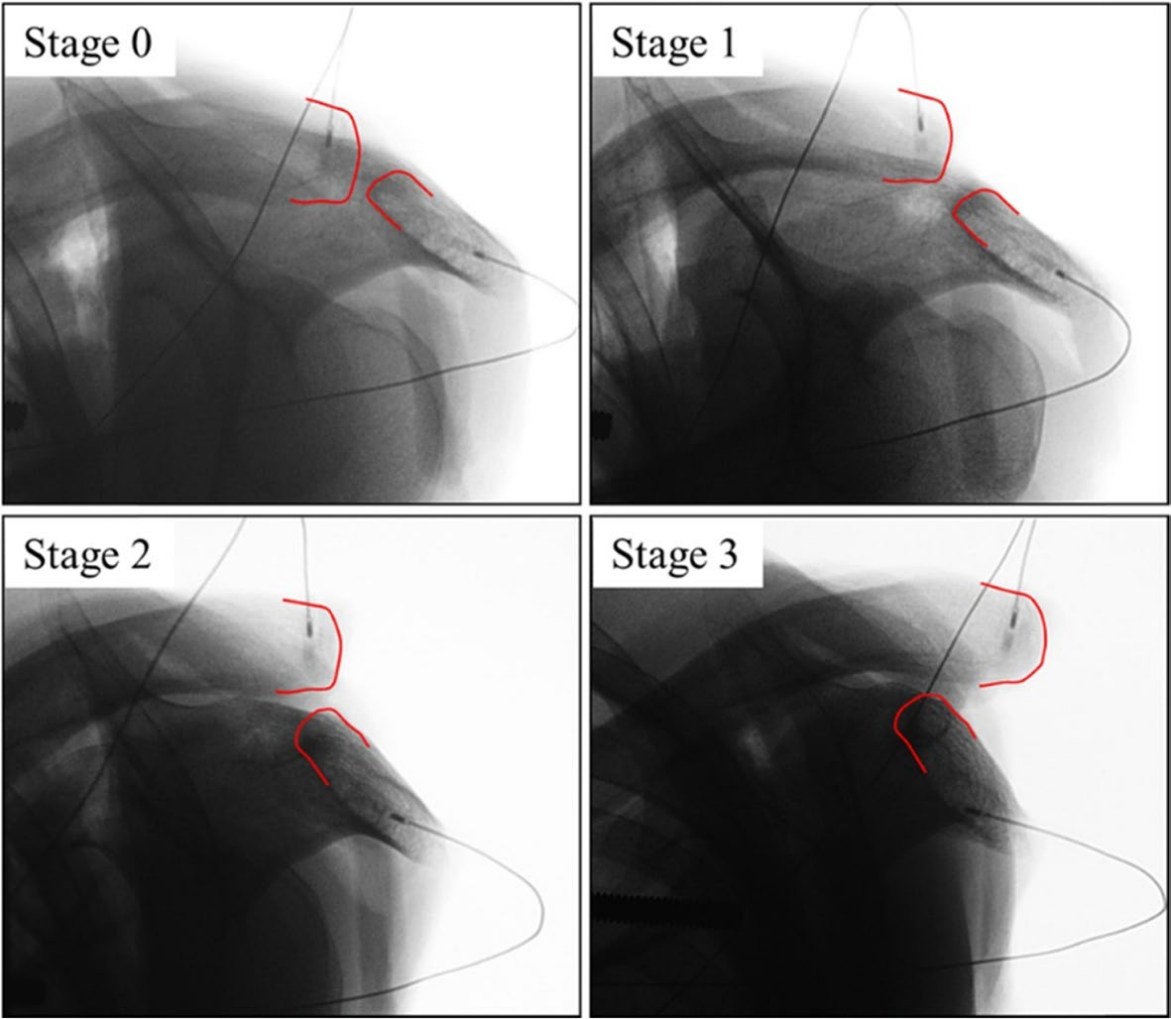
Cross-body adduction view in every stage. The distal end of the clavicle and acromion in the acromioclavicular joint are marked with red lines. The sensors are in the clavicle and acromion

The specimens were firmly fixed on a customized wooden jig with external fixators (Orthofix®; Japan Medicalnext Co., Ltd., Osaka, Japan). Five fully threaded stainless rods with a diameter of 6 mm were inserted into the 2^nd^ and lower cervical vertebraes, upper and lower thoracic vertebraes, and lower sternum. The rods were connected to external fixators (Fig. 1). The displacement of the distal end of the clavicle relative to the acromion was measured using an electromagnetic tracking device (trakSTAR™; Ascension Technology Corporation, Shelburne, VT, USA). Sensors were inserted into the distal end of the clavicle and acromion. The proper location of the inserted sensors was verified with AP radiographs (BV Pulsera; Philips, Best, Netherlands) (Fig. 2).

### Sectioning the distal clavicle stabilizers

**Fig. 3:**
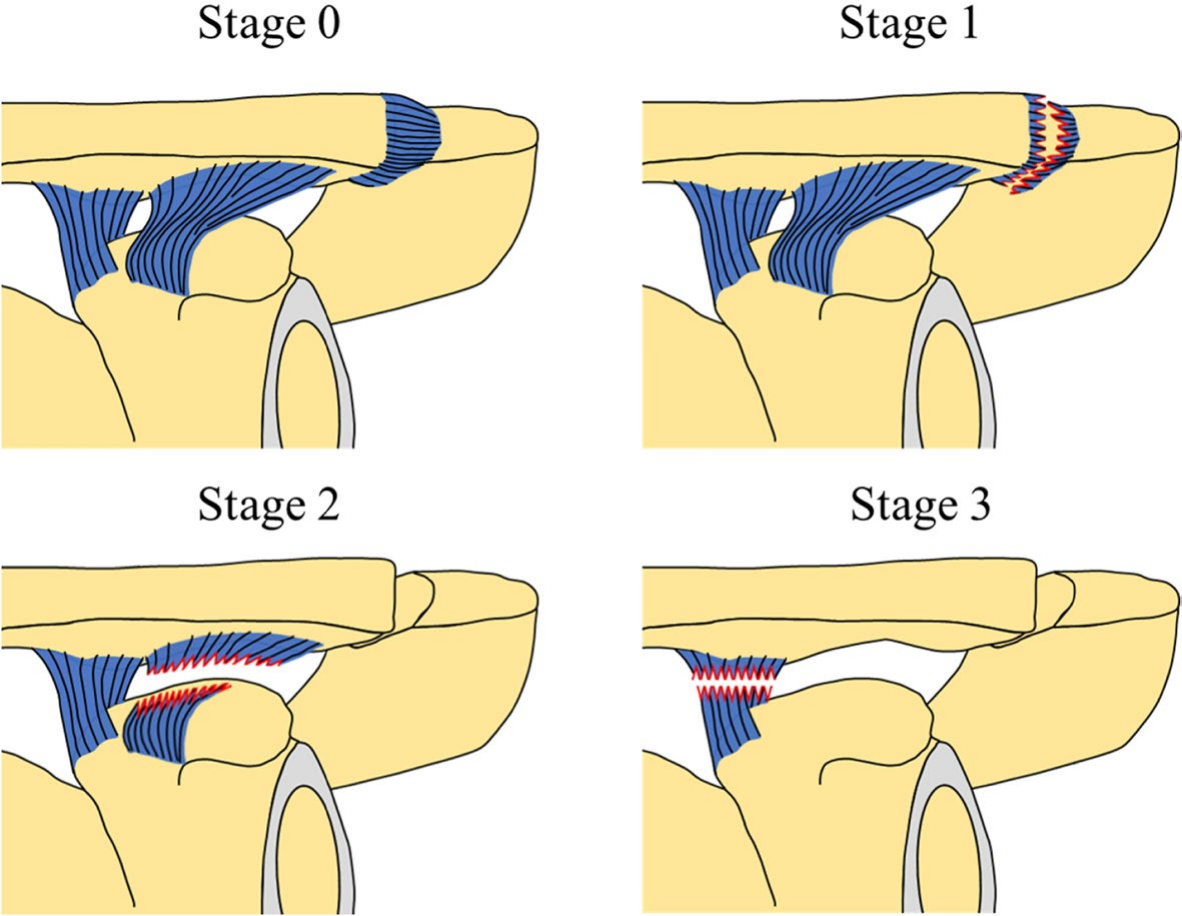
Simulation of AC joint dislocation. We simulated AC joint dislocation by sequential resection of AC ligament, AC joint capsule, trapezoid ligament, and conoid ligament in the following order of stages. Stage 0: The acromioclavicular and coracoclavicular ligaments and the acromioclavicular joint capsule were intact. Stage 1: The acromioclavicular ligament, acromioclavicular joint capsule, and disc were sectioned. Stage 2: The trapezoid ligament was sectioned. Stage 3: The conoid ligaments was sectioned. The sectioned ligaments in each stage are indicated by jagged lines

We resected the AC, trapezoid and conoid ligaments sequentially, and simulated AC joint dislocation model (Fig. 3). Sectioning stages were defined as follows. Stage 0: the AC and CC ligaments and the AC joint capsule were intact; stage 1, the AC ligament, AC joint capsule, and disc were sectioned; stage 2, trapezoid ligament were sectioned; and stage 3, conoid ligaments were sectioned. The trapezius and deltoid muscle were incised parallel to the AC joint when the AC ligament and joint disc were removed. When resecting the trapezoid and conoid ligaments, the deltoid muscle was incised in the direction of the muscle fibers, and the ligaments were resected after clearly viewing them. Ligaments were sectioned according to previous biomechanical studies [16, 17, 19].

### Loading testing and data acquisition

**Fig. 4:**
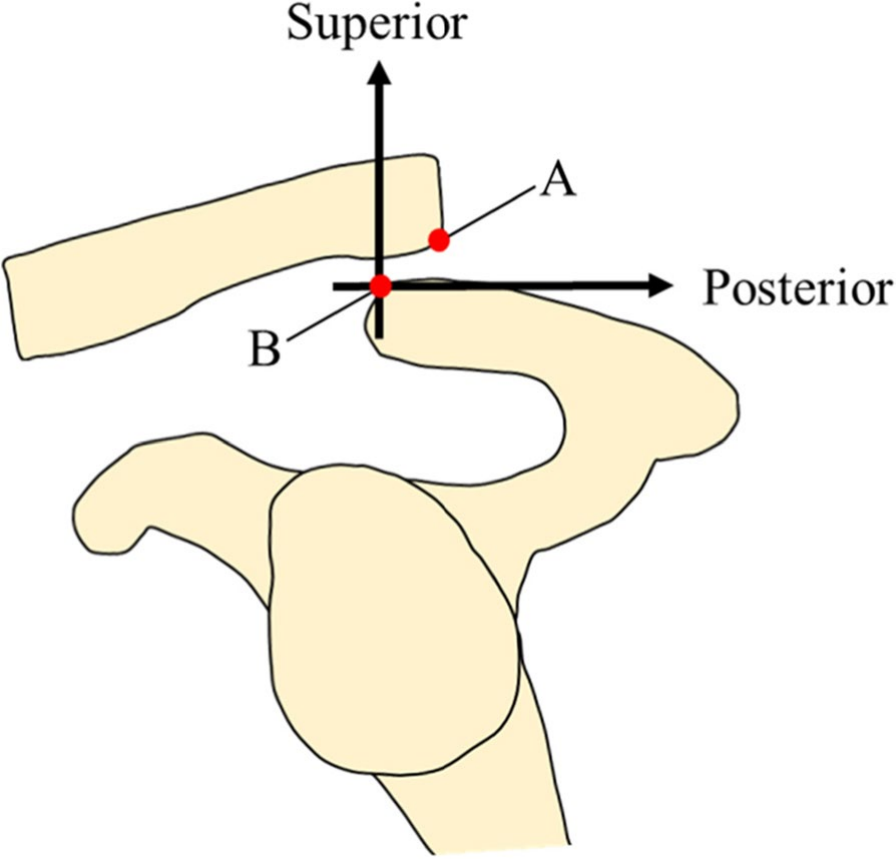
Point A: Inferior edge of the clavicle in the acromioclavicular joint. Point B: Superior edge of the acromion in the acromioclavicular joint. Clavicle overriding on the cross-body adduction view was defined as the superior and posterior displacement of point A compared to that of point B

Stress was added to the AC joint by pulling the cord that was passed through the humerus. An examiner elevated the upper limb to 90° in the sagittal plane and adducted the upper limb manually until the maximum adduction angle was acquired in the horizontal plane, the cross-body adduction radiography was also performed in this position. Using this imaging technique, we assessed the degree to which the clavicle overlapped the acromion because of anteromedial scapula translation. Clavicle overriding on the cross-body adduction view was defined as the superior or lateral displacement of the inferior edge of the clavicle in the AC joint compared to the that of superior edge of the acromion in the AC joint (Fig. 4).

We defined the direction parallel to the AC joint is the X-axis, perpendicular to the AC joint is the Y-axis, and perpendicular to the ground is the Z-axis. The magnitude of the displacement was measured in each direction. In the horizontal adduction position, the direction of the AC joint changed, as demonstrated by the electromagnetic tracking device; yet, we could not measure the horizontal translation of the AC joint. We predicted posterior instability by determining whether the distal clavicle overrode the acromion [20]. Displacement was measured in the Z-axis by calculating the difference between the values of both sensors in the acromion and the distal end of the clavicle. Values in stage 0 were used as control values.

### Statistical analysis

Displacement magnitudes between each stage were compared using a one-way analysis of variance, and post-hoc tests were performed using the Tukey–Kramer method. *P*-values of < 0.05 were considered statistically significant. Statistical analysis was performed using SPSS for Windows version 22.0 (IBM Corp., Armonk, NY, USA).

## Results

### Superior displacement

**Fig. 5:**
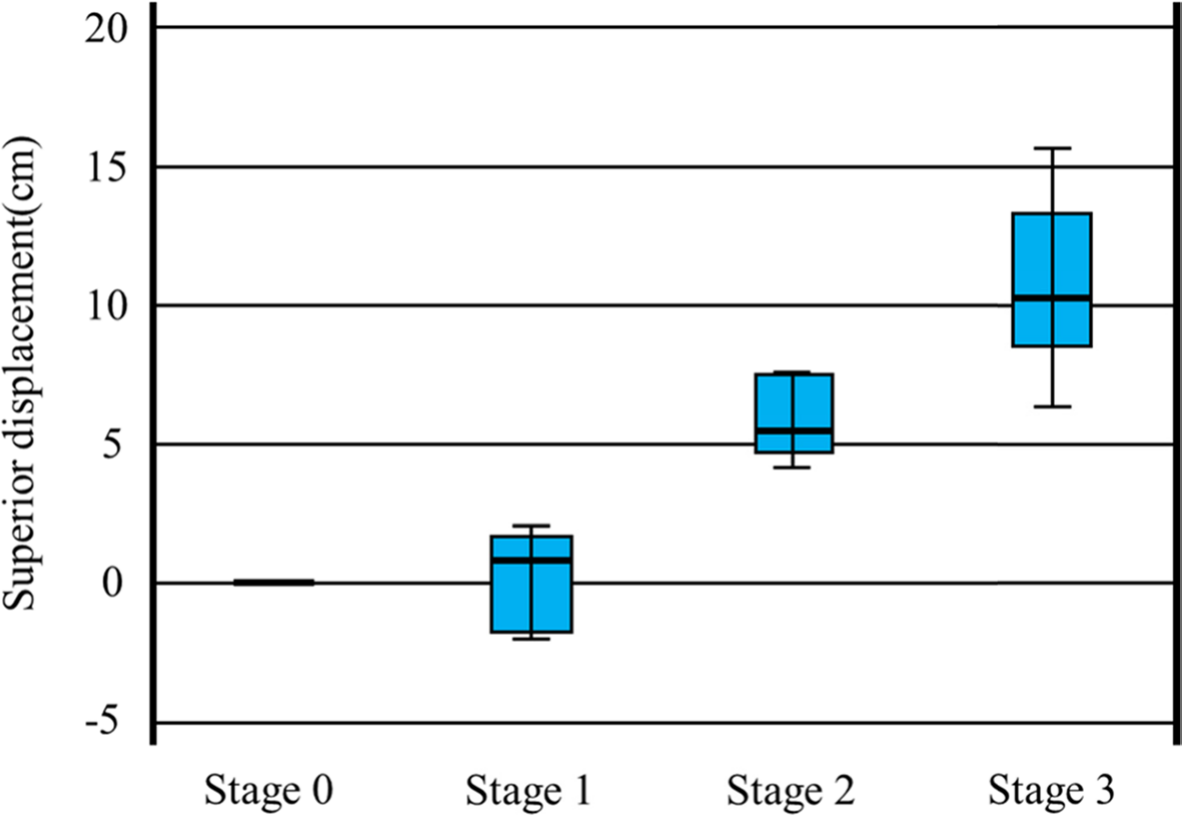
Superior displacement in each stage. There is no statistically significant difference between stages 0 and 1 (*P* = 0.997); however, displacement increased significantly between stages 0 and 2 (*P* = 0.001), between stages 0 and 3 (*P* < 0.001), between stages 1 and 2 (*P* = 0.001), between stages 1 and 3(*P* < 0.001), and between stages 2 and 3 (*P* = 0.026)

There was no statistically significant difference between stages 0 and 1 (*P* = 0.997); yet, displacement increased significantly between stages 0 and 2 (*P* = 0.001), between stages 0 and 3 (*P* < 0.001), between stages 1 and 2 (*P* = 0.001), between stages 1 and 3(*P* < 0.001), and between stages 2 and 3 (*P* = 0.026) (Fig. 5).

**Fig. 6:**
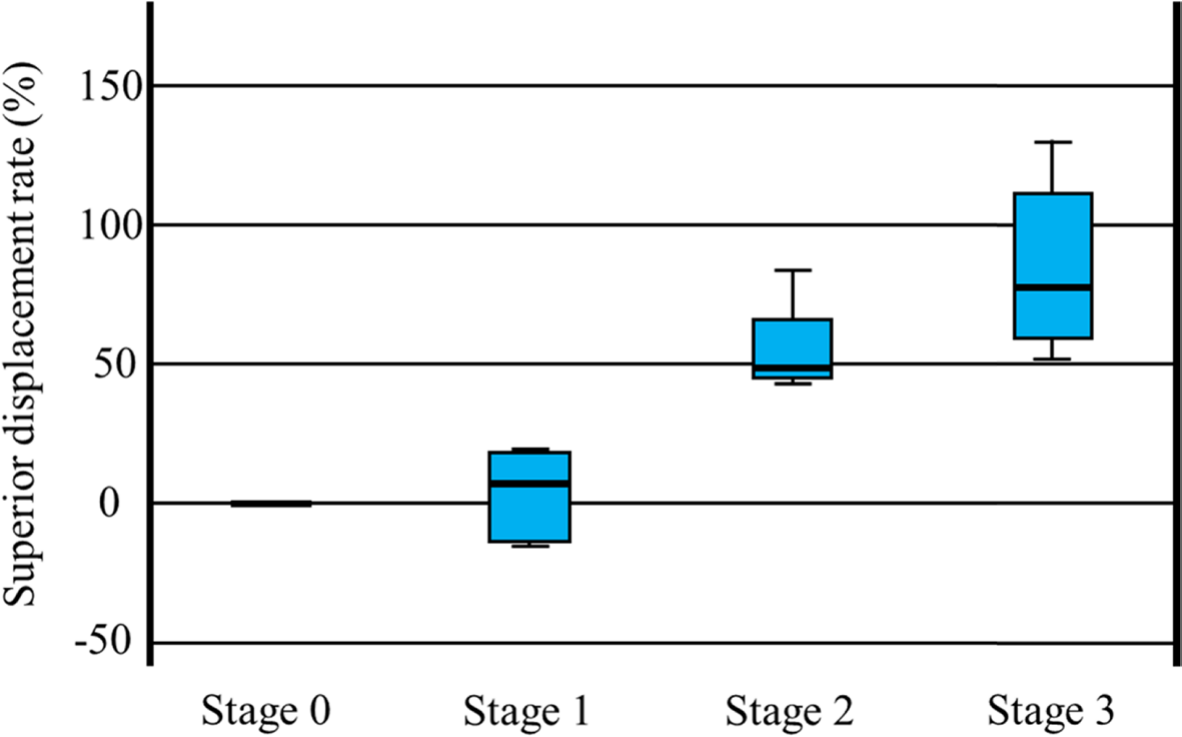
Superior displacement rate in each stage. There is no statistically significant difference in the rate of displacement between stages 0 and 1 (*P* = 0.980) and between stages 2 and 3 (*P* = 0.076), yet the displacement rate increased significantly between stages 0 and 2 (*P* < 0.001), between stages 0 and 3 (*P* < 0.001), between stages 1 and 2 (*P* = 0.001), and between stages 1 and 3 (*P* < 0.001)

There was no statistically significant difference in the rate of displacement between stages 0 and 1 (*P* = 0.980) and between stages 2 and 3 (*P* = 0.076) yet the displacement rate increased significantly between stages 0 and 2 (*P* < 0.001), between stages 0 and 3 (*P* < 0.001), between stages 1 and 2 (*P* = 0.001), and between stages 1 and 3 (*P* < 0.001) (Fig. 6).

### Distal clavicle overriding the acromion on cross-body adduction view

The distal clavicle did not override the acromion in stages 0 and 1; yet, five of six distal clavicles (83%) overrode the acromion in stage 2, and all distal clavicles overrode the acromion in stage 3 (Fig. 4, Table 1).

**Table 1:** Superior AC joint displacement and distal clavicle overriding in each sectioning stage on the cross-body adduction view

	Amount of Displacement, mm^a^	% of displacement^b, c^	Distal clavicle overriding
Stage 0	0	0	0/6
Stage 1	0.3 ± 0.7	4.2 ± 15.0	0/6
Stage 2	6.5 ± 1.1	56.2 ± 15.8	5/6
Stage 3	10.7 ± 1.4	84.3 ± 30.6	6/6

^a^ Data are reported as mean ± standard deviations

^b^AC joint height = 11.3 ± 1.6

^c^Calculated as [(change in superior displacement from stage 0)/(AC joint height)] × 100. Data are reported as mean ± standard deviations

## Discussion

The most important findings of this study are that sectioning the AC and trapezoid ligaments causes AC joint instability and that the clavicle can override the acromion on cross-body adduction view regardless of conoid ligament sectioning. ISAKOS [8] suggested distinguishing two variations of type III Rockwood injury using a cross-body adduction view. Zumstein et al. [20] reported the use of the acromial center line spanning to the dorsal clavicle (AC-DC) to assess vertical displacement and the use of the glenoid center line spanning to the posterior clavicle (GC-PC) to assess horizontal displacement; performing these evaluations in a single Alexander view is recommended to guide the appropriate management of AC joint dislocations. Karargyris et al. [21] reported that AC-DC measurement seemingly represents a more realistic AC joint injury magnitude than the CC distance. Increasing attention has been given to the relationship between Alexander view evaluation and AC joint stability [20, 21]. In this study, we referred to radiological AC joint dislocation evaluation and investigated the relationship between ligament injury, cross-body adduction view, and AC joint instability. AC joint instability in both superior and posterior directions will make the distal clavicle override the acromion [20].

Regarding AC joint superior stability, Fukuda et al. [14] described the contributions of individual ligaments to joint stability by performing biomechanical loading experiments using fixed displacements and sequential ligament sectioning. At small displacements, the AC ligament primarily restraints the superior direction of the AC joint, and at large displacements, the conoid ligament primarily restraints the superior direction of the AC joint. Kurata et al. [16] reported that the AC ligament significantly contributes to AC joint stability in the superior direction and AC joint displacement > 50% may occur with isolated AC ligament injury in biomechanical loading experiments.

Regarding posterior stability, Fukuda et al. [14] reported that the AC ligament primarily restraints the posterior direction of the AC joint, and Dawson et al. [22] and Dyrna et al. [23] biomechanically investigated the kinematics of the AC joint and reported that the AC ligament strongly contributes to the horizontal stability of the AC joint. Oki et al. [17] conducted a biomechanical investigation using whole cadaver models and evaluated the function of the AC and CC ligaments in scapular and clavicular motions during humerothoracic motions and found that the AC ligament restraints clavicular retraction and that the trapezoid ligament restraints scapular internal rotation during horizontal plane adduction. Debski et al. [18] reported that the trapezoid ligament plays an important role in the posterior direction of the AC joint. Kurata et al. [16] reported that sectioning the trapezoid ligament after sectioning the AC ligament causes AC joint instability.

According to a previous studies that examined superior and horizontal AC joint stability [14, 16–18, 22, 23], superior AC joint stability relies on the AC and conoid ligaments, whereas posterior AC joint stability relies on the AC and trapezoid ligaments. The results of this study revealed that five of the six clavicles overrode the acromion at stage 2, regardless of conoid ligament sectioning. Although the average superior displacement of the five clavicles was not large (average, 6.7 mm), sectioning the AC and trapezoid ligaments alone may cause AC joint instability enough to override the clavicle on the acromion in the horizontal adduction position. Our results suggest that there are variations of CC ligament injuries in type IIIB (unstable) injury of AC joint dislocation. Diagnosis of AC joint dislocation should not only be based on the radiographic appearance of the AC joint [16, 25]. Recently, there are many reports about evaluation of the AC joint dislocation using magnetic resonance imaging and the accuracy of diagnosing ligament injuries [24, 25].

This study has some limitations. First, lateral-to-medial ligament sectioning was performed according to previous studies, and the sequences of ligament sectioning may differ from the order of ligament injuries in actual patients with AC joint dislocation. Second, this study used the cadavers of old people; yet, AC joint dislocation is common among young people. Third, the deltoid muscle is assumed to the stabilizer of the AC joint [15, 26]. Therefore, when resecting the trapezoid and conoid ligaments, the deltoid muscle was incised in the direction of muscle fibers. Fourth, the direction of the AC joint changed, and we did not evaluate the horizontal translation of the AC joint in the horizontal adduction position. Fifth, we used a limited number of specimens. Sixth, sectioning was not randomized; the remaining ligaments were stressed more due to the repeated application of loading forces for each stage. Seventh, spine fixation was required to evaluate one side scapulothoracic motion, and only one shoulder could be used from one specimen in this experimental model.

## Conclusion

In the current fresh-frozen cadaver models, we found that AC and trapezoid ligament sectioning caused AC joint instability and that the clavicle can override the acromion on cross-body adduction view without conoid ligament sectioning. Accordingly, CC joint injury variations may exist in AC joint dislocation cases, and these injuries might override the acromion on cross-body adduction view. Further investigations will be required to evaluate the degree of AC joint dislocation.

## Data Availability

The datasets used during the present study are available from the corresponding author upon reasonable request.

## Abbreviations

AC: Acromioclavicular
CC: Coracoclavicular
ISAKOS: International Society of Arthroscopy, Knee Surgery and Orthopaedic Sports Medicine
